# Effects of Royal Jelly Administration on Lipid Profile, Satiety, Inflammation, and Antioxidant Capacity in Asymptomatic Overweight Adults

**DOI:** 10.1155/2019/4969720

**Published:** 2019-06-13

**Authors:** Ana Petelin, Saša Kenig, Rok Kopinč, Matjaž Deželak, Maša Černelič Bizjak, Zala Jenko Pražnikar

**Affiliations:** ^1^University of Primorska, Faculty of Health Sciences, Polje 42, SI-6310 Izola, Slovenia; ^2^Medex d.o.o., Linhartova 49A, SI-1000 Ljubljana, Slovenia

## Abstract

**Objectives:**

Obesity and overweight are chronic disorders of multifactorial origin that are characterized by high oxidative status and by chronic activation of macrophages in peripheral tissues. Effective therapeutic approaches to lower inflammation and oxidative stress are currently of general interest. Royal jelly (RJ) is a functional food with a broad range of pharmacological activities, mainly used by healthy individuals or borderline patients to protect themselves against disease onset. The objective of this randomized, double-blind, placebo-controlled trial was to investigate the effects of RJ supplementation on metabolic profile and oxidative and inflammatory parameters in asymptomatic overweight adults, considered at an early stage of developing metabolic syndrome.

**Material and Methods:**

The experimental group (n=30) was given RJ and the control group (n=30) was provided with a placebo for eight weeks. Anthropometric, biochemical parameters, biomarkers of oxidative stress, and inflammation were assessed at baseline, after 4 and 8 weeks of the intervention, and after additional 2 weeks of follow up.

**Results and Conclusion:**

Compared with the placebo, RJ supplementation demonstrated a statistically significant decrease in total cholesterol (6.7%;* p*=0.041) and inflammatory marker C-reactive protein (19%;* p*=0.027), whereas significant increases were observed in anti-inflammatory marker adiponectin (34%;* p*=0.011), endogenous antioxidants bilirubin (35%;* p*=0.002) and uric acid (5%;* p*=0.018), total antioxidant capacity in serum (54%;* p*=0.005), and leptin (17%;* p*=0.025). The present study demonstrated positive effects of RJ administration on lipid profile, satiety, inflammation, and antioxidant capacity in overweight adults. Therefore, our study supports the benefits of RJ supplementation for the improvement of human health.

## 1. Introduction

The incidence of obesity has been rapidly increasing, and this condition has become a major public health threat, since it is strongly linked with increased risk for several diseases including type 2 diabetes, cardiovascular disease, cancer, and brain aging [1, 2]. Obesity and overweight are chronic disorders of multifactorial origin that are characterized by high oxidative status [3] and by chronic activation of macrophages in peripheral tissues [4, 5]. Activated macrophages yield unresolved inflammation in peripheral organs including the adipose tissue [4, 6] and liver [7]. Thus far, molecular mechanisms underlying obesity and obesity‐related metabolic disorders have not been fully clarified, and effective therapeutic approaches to lower inflammation and oxidative stress are currently of general interest [8].

In recent years, the consideration of natural products as anti-inflammatory and antioxidative treatments has grown worldwide. Moreover, natural products are easily obtained and are relatively safe. Royal jelly (RJ) is a mixture of natural products secreted from the hypopharyngeal and mandibular glands of nurse honeybees (*Apis mellifera*). It is essential for queen reproduction and larvae development in honeybee colonies. RJ is now widely used as a dietary supplement and in cosmetic products [9] and is as a functional food of interest for the improvement of human health.

RJ contains a specific combination of water, sugars, proteins, and lipids and approximately 90% of RJ lipids are free fatty acids, containing 8–12 carbon atoms that are usually in either hydroxyl or dicarboxylic form [10]. 10-hydroxy-2-decenoic acid (10-HDA), most known bioactive component of RJ, plays important roles in various biological activities, including inflammation and oxidative stress [11]. Among the proteins, majority of them are categorized as Major Royal Jelly Proteins (MRJPs), which are considered to be key factors in honey bee queen development [12]. RJ has been demonstrated to exhibit a broad range of pharmacological activities* in vitro* and in some human studies, including promotion of cell growth and wound healing [13, 14], antibacterial and antifungal activities [15, 16], hypocholesterolemic effects [17], antitumor activity [18, 19], vasodilative and antihypertensive activity [20], antioxidant activity [21–23], and anti-inflammatory activity [24, 25]. To date, most studies have been performed* in vitro *and in selected groups of patients; none of the study has thus far been designed for asymptomatic adults with low-grade systemic inflammation.

As effective therapeutic approaches to lower inflammation and oxidative stress are currently of general interest and considering the potential beneficial effects of RJ intake, the purpose of the present study was to investigate the effects of RJ consumption in comparison to placebo on metabolic profile, inflammatory markers, and antioxidant capacity in asymptomatic overweight adults, considered at an early stage to develop metabolic syndrome.

## 2. Methods

### 2.1. Composition of RJ and Placebo Capsule

Experimental RJ capsules and placebo capsules were provided by Medex. According to the manufacturer, each RJ capsule contained 333 mg of organic lyophilised RJ, standardized to a minimum of 4% 10-HDA, which corresponds to 1000 mg of fresh RJ. In adittion RJ capsule contained also less than 7% of moisture, 8-19% of lipids, 24-41% of proteins, and pH between 3.4-4.5. The placebo capsule contained an equal amount of rice starch. Both experimental RJ and placebo capsules appeared similar to each other in terms of colour, flavour, size, and shape to make sure that they cannot be distinguished.

### 2.2. Clinical Study

#### 2.2.1. Study Participants

**Figure 1:**
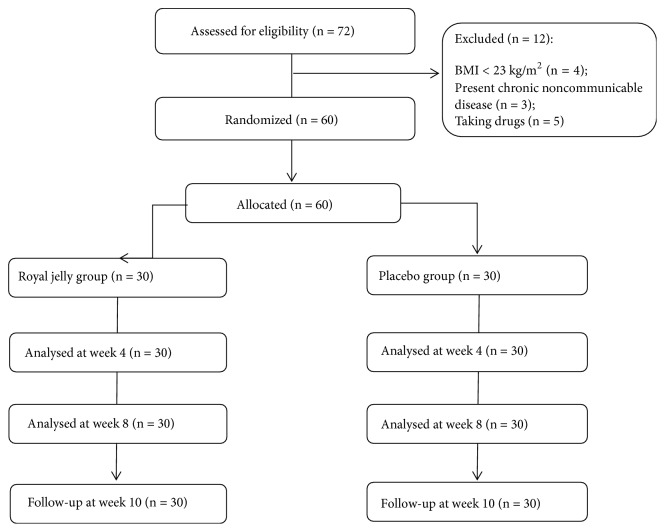
Recruitment flow chart of healthy asymptomatic overweight adults.

Seventy-two asymptomatic overweight adults aged 25-50 were recruited into the study (Figure 1). The inclusion criteria were BMI between 23 and 30, no more than 3% change of body mass within the last three months, and age between 25 and 50 years. The exclusion criteria were cardiovascular, gastrointestinal, or liver disease, type 2 diabetes mellitus (T2DM), taking medications for lipid disorders, anti-inflammatory drugs (NSAID), or nutritional supplements, and being pregnant or lactating. The Caucasian male and female volunteers were recruited through advertisement posted on internet forums, sent via e-mail lists and published in local newspapers. Twelve participants did not meet the inclusion criteria; therefore, the final analytical sample was sixty volunteers, thirty in experimental and thirty in control group. This trial was conducted in accordance with the Declaration of Helsinki, 2008. Eligible participants were included after we received their written informed consent. The study protocol was approved by the Slovenian National Medical Ethics Committee (No. 0120-207/2018/4).

#### 2.2.2. Study Design

The study was randomized, double-blind, placebo-controlled trial. Sixty participants were randomly divided into 2 groups, the experimental RJ group and the placebo group. Randomization with stratification was performed using simple software. Stratified variables were gender and age. The study was conducted at the University of Primorska, Faculty of Health Sciences, between April and July 2018. The entire study consisted of 8 weeks of administration period (intervention), during which subjects in the experimental or placebo groups were prescribed to intake two capsules (333 mg/capsule) of lyophilised RJ or placebo every morning before breakfast, with two weeks of follow-up without any supplementation. A questionnaire was given to each participant at the beginning of the study and after 8 weeks, to report any adverse events. Anthropometric, appetite, and mood measurements were performed at baseline (initial), after 4 and 8 weeks during intervention and after 10 weeks (2 weeks follow-up) of RJ or placebo supplementation. In addition, fasting blood samples were also collected for biochemical assays at the same time points. Dietary intake of participants was followed during the study by using 24-h recall. All participants received the same instructions. They were free to consume their own diet and were encouraged to avoid any alterations to their normal diet and physical activity except for the required encapsuled product during 8 weeks intervention phase. All participants refrained from taking antibiotics and other nutritional supplements or other drugs. An expert dietitian carefully collected in-person list of all foods and beverages, using food models. Dietary data was analysed using the Open Platform for Clinical Nutrition (OPEN) accessible through the website http://opkp.si/. Compliance with the supplementation protocol was supervised by a researcher who contacted the subjects once a week. Each subject was required also to return the original botle of their respective supplement for capsule counts.

#### 2.2.3. Appetite Measure

Appetite and other sensations were assessed using Simplified nutritional appetite questionnaire (SNAQ) [26] and Visual analogue scale (VAS) [27]. The SNAQ is self-report appetite assessment tool, with a short 4-item single-domain questions. The participants were asked to answer questions assessing their appetite, feelings of fullness, feelings of food tastes, and number of meals eaten in the last week. Responses are scored by using a 5-point, verbally labeled, Likert-type scale. The total SNAQ score is the sum of scores on the four questions, with lower scores indicating deterioration in appetite. Possible scores range from 4 (low) to 20 (high). The VAS for appetite measurement and desire to eat consisted of a series of 100 mm lines anchored extreme appetite perceptions on both ends of each line (e.g., not at all hungry-very hungry). They were used to assess hunger and desire to eat (e.g., to eat something sweet or salty: very much-not at all). Subjects were requested to make a vertical mark on a line (e.g., 7-point equal interval) that the best matched how they were feeling in the last few days. This scale was anchored at 0 (not at all hungry) to 7 (very hungry). Each score was determined by measuring the distance from the left side of the line to the mark. Average subjective appetite value was calculated as the average of the scales for all visual scales. Finally, evaluation of the appetite was calculated as the average of the two self-reported appetite values.

#### 2.2.4. Mood Measure

Mood was measured using the Positive and Negative Affect Schedule (PANAS) questionnaire [28]. The PANAS is a 20-item scale. Ten items assessed positive affect (PA) or mood (e.g., excited, active, and enthusiastic), and 10 items measured negative affect (NA) or mood (e.g., irritable, distressed, and nervous). Watson et al. stated that high PA is a state of high energy, full concentration, and pleasurable engagement, whereas low PA is characterized by sadness and lethargy. Negative affect (NA) is a general dimension of subjective distress and unpleasable engagement, with low NA being a state of calmness and serenity'. Subjects were instructed to respond to the PANAS questionnaire on the basis of how they felt at that moment and indicate “to what extent do you feel this way right now” [28]. Their answers ranged from 5 (extremely) to 1 (very slightly or not at all). Authors report acceptably high alpha reliabilities, ranging from 0.86 to 0.90 [28].

#### 2.2.5. Anthropometric Measurements

All measurements (at baseline, after weeks 4, 8, and 10) were performed following an overnight fast between 7 am and 9 am in standardized conditions by the same examiner. Body weight was measured in light clothing without shoes, to the nearest 0.1 kg and height to the nearest 0.1 cm, using a Leicester Height Measure (Invicta Plastics Limited, Oadby, England). BMI was calculated as weight (kg) divided by height (m) squared. Body composition (total percentage body fat (BF), fat-free mass (FFM), visceral fat, total body water (TBW), and phase angle) was assessed using bioelectrical impedance analysis (BIA) Tanita MC-980MA (Maeno-cho, Japan) and dedicated software (GMON Pro-Tanita).

#### 2.2.6. Blood Parameters

Venous blood samples were collected at baseline and after weeks 4, 8, and 10 following an overnight fast in 6 ml vacuum test tubes (Beckton-Dickinson, Rutherford, USA). Serum was immediately separated, frozen, and stored at −80°C until subsequent analysis. Serum concentrations of glucose, triacylglycerols (TAG), total cholesterol, low density lipoprotein (LDL), high density lipoprotein (HDL), C-reactive protein (CRP), bilirubin, and uric acid (UA) were measured using Cobass reagents and performed on a Cobass c111 analyzer (Roche). Serum concentrations of adiponectin, leptin, cortisol, neuropeptide Y (NPY), and brain-derived neurotrophic factor (BDNF) were determined in duplicate on a microplate reader (Tecan, Männedorf, Switzerland) using human ELISA Kits for adiponectin and leptin (BioVendor, Czech Republic), NPY, BDNF (both EMD Millipore Corporation, USA), and cortisol (IBL international GMBH, Germany). Assay sensitivity was 10 pg/ml for adiponectin, 0.2 ng/mL for leptin, 3 ng/dL for cortisol, 2 pg/mL for NPY, and 15 pg/mL for BDNF. Assays interassay and intraassay CVs were typically <10%. The total antioxidant capacity of serum samples was measured by Photochem instrument (Analytic Jena, Jena, Germany) using ACW kits (Water Antioxidant Capacity) to detect the activity of hydrophilic compounds. The ultra-sensitive photochemiluminescence assay was carried out with the procedure described by Popov and Lewin [29].

#### 2.2.7. RNA Extraction, Quantitative Real Time PCR, and Data Analysis

Peripheral blood mononuclear cells were isolated with Histopaque-1077 reagent (Sigma-Aldrich) from 2 mL of fresh blood samples collected into EDTA vacutainers (BD) at baseline and after week 8. RNA was isolated using TRIzol reagent (Thermo-Fisher Scientific) following manufacturer's instructions. Quality was examined spectrophotometrically and two *μ*g were transcribed to cDNA with cDNA Archive kit (Applied Biosystems). For the quantitative RT-PCR reactions, QuantStudio® 5 Real-Time PCR System and SYBR Green master mix were used. The primers with the following IDs were selected from the PrimerBank [30]: 48762945c1 for superoxide dismutase 1, 260436906c3 for catalase, 41406081c1 for glutathione peroxidase 1, 305410788c1 for glutathione reductase, 196049379c2 for 3-hydroxy-3-methylglutaryl-CoA reductase, 148298676c2 for 3-hydroxy-3-methylglutaryl-CoA synthase 1, and 307775415c1 for low-density lipoprotein receptor. 18S rRNA was used as internal control. PCR conditions were 50°C for 2 min, 95°C for 10 min, and 40 cycles of 95°C for 15 s and 60°C for 1 min. The data were analyzed by the ΔΔCt algorithm. Melting curves were inspected to ensure primer specificity.

#### 2.2.8. Statistics

Sample size was calculated considering power of 90% with a 2-sided test, with *α*=0.05 (type I error) to detect a 5% difference in serum glucose between two groups. The number of subject needed was 20 per group. However, we set the enrollment target at 25 participants.

All results were explicated as a mean ± standard deviations (SD) or percentage. The normality of variables was tested by the Kolmogorov-Smirnov test. Two-way analysis of variance (ANOVA) was used to examine the effect of two independent variables on one continuous dependent variable, “time”, and “treatment”. In addition, paired sample was used to compare the measered parameters between two time points. Independent sample t-test was utilized to compare the differences between the experimental and placebo group. Statistical analyses were performed with the help of computer software–statistical package for the social sciences (SPSS) version 23.0 (IBM Inc., Chicago, IL). All statistical outcomes with p values less than 0.05 (p<0.05) were recognized as statistically significant.

## 3. Results

### 3.1. Baseline Participant Characteristics

Sixty participants were enrolled in this randomized, double-blind, placebo-controlled trial and assigned to either the placebo (n=30) or the RJ group (n=30).  Table 1 shows the baseline characteristics of participants in both groups. There were no significant differences in means of age, gender, body fat, BMI, total cholesterol, and glucose between the two groups. Energy intake and macronutrient composition of the diets did not differ between the two groups at baseline and did not change during the intervention period (Table 2). No severe adverse effects were observed. According to the count of the recalled capsules at the end of the intervention, compliance was very good. The rates of capsule intake were 97% and 94% in the RJ and placebo group, respectively.

### 3.2. Effect of RJ on Anthropometric Parameters

Regarding anthropometric parameters, significant effects of treatment and time were found for phase angle (F=29.4, p=0.010, and *η*^2^=0.90; F=7.47, p=0.041, and *η*^2^=0.470). Additionally, significant effect of time was observed for % of body fat (F=11.2, p=0.027, and *η*^2^=0.627) and a significant effect of treatment was found for BMI (F=35.6, p=0.008, and *η*^2^=0.918).  Table 3 shows anthropometric parameters before and after the intervention. The baseline anthropometric parameters did not differ significantly between the two groups. In placebo group, no statistically significant differences in body composition were observed in the whole period of the clinical trial (Table 3). On the other hand, continuous use of RJ for 4 weeks resulted in statistically significant decrease in body fat and increase in phase angle as compared with the initial values in RJ group (Table 3). After 8 weeks of the use of RJ, only phase angle remained significantly higher from the baseline. The significance of the difference between the change from baseline on RJ vs. placebo at week 8 was observed only for phase angle (p=0.048) (Table 3). Thus, RJ consumption induced some physiological changes of the subjects during the experimental period and these anthropometrical changes were also noted at follow-up period. Moreover, the significance of the difference between the change from baseline on RJ vs. placebo at follow-up was observed also for BMI (P <0.05).

### 3.3. Effects of RJ on Biochemical Parameters

Significant effects of treatment and time were found for total serum cholesterol (F=38.4, p=0.007, and *η*^2^=0.975; F=388, p<0.001, and *η*^2^=0.998) and LDL cholesterol (F=14.9, p=0.026, *η*^2^=0.937; F=324, p=0.001, and *η*^2^=0.979), respectively. Additionally, a significant effect of treatment was observed for glucose (F=6.5, p=0.048, and *η*^2^=0.683) and TAG (F=11.7, p=0.033, and *η*^2^=0.753). Measured biochemical parameters are summarized in Table 4. The baseline concentrations of lipids and glucose did not differ significantly between the two groups. Total serum cholesterol and triacylglycerol levels were significantly lower at week 4 than at baseline in the RJ group (Table 4). Although TAG values decreased at week 4 from baseline, there was no significant difference between the two groups at week 4. At week 8, total serum cholesterol in the RJ group was still significantly lower than at baseline and a significant decrease compared with those in the placebo group was observed (p=0.041), whereas TAG returned to baseline. Moreover, fasting glucose in the RJ group was significantly lower at week 8 than at baseline, but there was no significant difference in changes between the two groups. Additionally, at follow-up, the experimental group showed significant decrease (p<0.05) in the total and LDL cholesterol levels by 7.8% and 6.2% with initial period and a significant decrease compared with those parameters in the placebo group (Table 4). The HDL cholesterol has no significant differences between baseline and each point of the clinical intervention, and there was no significant difference between the two groups (Table 4). In the placebo group, the levels of total, LDL, and HDL cholesterol, triacylglycerols, and fasting glucose were not changed.

### 3.4. Changes in Inflammatory Markers and Antioxidants

**Figure 2:**
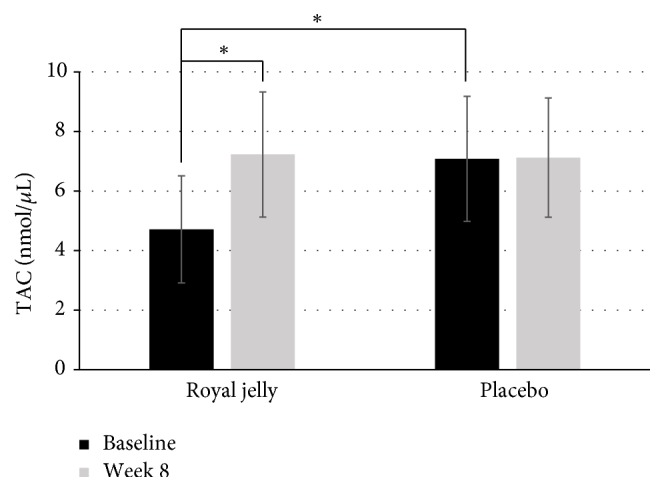
*Total antioxidant capacity (TAC) in asymptomatic subjects before and after intervention. ∗* p-value denotes significant (p<0.05) difference between serum TAC levels.

Significant effects of treatment and time were found for adiponectin (F=961.5, p=0.001, and *η*^2^=0.998; F=239.3, p=0.004, and *η*^2^=0.996), bilirubin (F=17.5, p=0.023, and *η*^2^=0.867; F=9.3, p=0.034, and *η*^2^=0.778) and CRP (F=21.2, p=0.016, and *η*^2^=0.905; F=10.1, p=0.032, and *η*^2^=0.797), respectively. The results showed that, after supplementation with RJ (at week 8), the levels of bilirubin and adiponectin were greatly enhanced (p<0.05), while levels of CRP were significantly reduced. Anti-inflammatory marker adiponectin and inflammatory marker CRP remained significantly different also after follow-up period in RJ group, while levels of bilirubin returned to the baseline (Table 5). On the other hand, levels of bilirubin and uric acid decreased in placebo group. Due to different trend in mentioned parameters, significant differences between both groups were observed at week 8 (p=0.027 for CRP; p=0.011 for adiponectin; p=0.002 for bilirubin; p=0.018 for uric acid) and during follow-up. Overall, RJ supplementation for 8 weeks significantly decreased the levels of CRP, whereas levels of adiponectin, bilirubin, and uric acid (UA) were increased significantly compared with the placebo. Moreover, the RJ group at week 8 demonstrated a statistically significant higher TAC compared with the placebo group (Figure 2). However, the baseline concentrations of TAC and bilirubin differed significantly between the two groups (Table 5, Figure 2).

### 3.5. Changes in the Levels of Hormones

Significant effects of treatment and time were observed only for leptin levels (F=687.8, p=0.001, and *η*^2^=0.997; F=81.3, p=0.012, and *η*^2^=0.988). Supplementation with RJ resulted in a significant increase in leptin levels compared with those in the placebo group at week 8 (p=0.025) and at follow-up (p=0.029). Meanwhile, the levels of NPY, cortisol and BDNF contents were not different between and within groups (Table 6).

### 3.6. Changes in Mood and Appetite

Significant effects of treatment and time were found only for self-reported appetite perceptions (F=65.6, p=0.001, and *η*^2^=0.941; F=14.8, p=0.026, and *η*^2^=0.937). The results showed that, in RJ group, self-reported appetite perceptions were significantly reduced after 4 weeks of supplementation with RJ and a decrease in self-reported appetite was noted also after follow-up period. In addition, after 8 weeks of supplementation with RJ, the negative affect or mood was slightly reduced in RJ group, but no statistically significant changes were noted during the follow-up period. Meanwhile, the positive affect or mood was not changed neither in experimental nor in placebo group (Table 7).

### 3.7. Changes in Gene Expression after RJ or Placebo Supplementation

**Figure 3:**
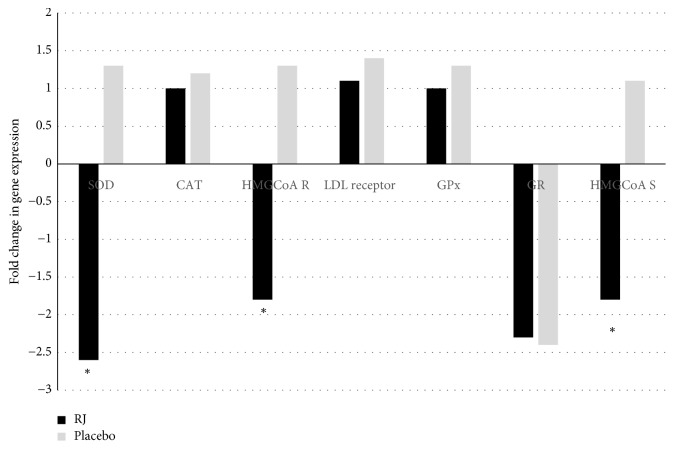
*Fold change in expression of seven genes after 8-week RJ or placebo supplementation relative to baseline.* Genes of oxidative stress and cholesterol metabolism pathways are reported, comparing RJ* versus* placebo:* SOD*: Superoxide Dismutase*; CAT*: Catalase;* GPx*: Glutathione Peroxidase;* GR*: Glutathione Reductase; LDL receptor;* HMGCoA R*: Hydroxymethylglutaryl-CoA reductase;* HMgCoA S*: Hydroxymethylglutaryl-CoA synthase.*∗* p-value denotes significant (p<0.05) difference between both groups (royal jelly vs. placebo).

We analysed seven genes at baseline and after 8 weeks of nutritional intervention. In particular, we focused on different expression levels of four genes of oxidative stress and three genes of cholesterol metabolic pathway. The different fold change levels were analysed for the condition RJ versus placebo. After data normalization, we identified differential gene expression levels for each condition, as shown in Figure 3. Our results showed a downregulation of superoxide dismutase (SOD) gene in RJ group compared to placebo group and a downregulation of hydroxymethylglutaryl-CoA reductase (HMgCoA Reductase) and hydroxymethylglutaryl-CoA synthase (HMgCoA Syntase) genes in RJ group compared to placebo group. Changes in expressions of other analyzed genes (*CAT,* LDL receptor gene,* GPx, *and* GR*) were not different between groups (RJ* versus* placebo) after intervention.

## 4. Discussion

RJ is a popular commercial product in the medicine, food, and cosmetic industry. In addition to being nutritive, RJ has several health promoting properties. The properties of RJ are multifactorial due to huge variability of bioactive substances and the variety of physiological processes it presumably affects [31]. Based on our main results, 666 mg of lyophilised RJ significantly reduced total serum cholesterol and CRP and significantly increased serum adiponectin, bilirubin, uric acid, TAC, and leptin after 8 weeks in asymptomatic overweight adults compared to placebo. The present results support its antioxidant, anti-inflammatory, and hypolipidemic activities, so far mostly studied in animal models and in selected groups of patients [32–34]. Additionally, our results propose effects of RJ on satiety, which is a new observation not yet reported in the literature.

Dyslipidaemia is one of the most common features of chronic noncommunicable diseases and different natural products that ameliorate lipid profile are of general interest [35]. In agreement with results of [34, 36, 37], the administration of RJ concomitantly significantly reduced the total and LDL cholesterol levels in the present study. Meanwhile, the levels of triacylglycerol's and HDL cholesterol were unaltered upon administration with RJ. This supports the known antihyperlipidemic effect of RJ, probably due to its active components, 10-HDA, and MRJP1, as pointed out by Nagaoka et al. [38]. In addition, similarly to Kamakura and Sataki [14] our results showed a downregulation of* HMgCoA Reductase *and* HMgCoA Synthase* genes in RJ group compared to placebo group, pointing out to reduced levels of the cholesterol biosynthesis enzymes. However, we are aware that changes in gene expression were analyzed in lymphocytes and not in the liver, where cholesterol biosynthesis occurs. On the other hand, changes in expression of LDL receptor gene were not different between RJ and placebo groups. Nevertheless, considering the beneficial effects of reduction of the coronary mortality rate by ameliorating the lipid profile, an approximately 6-7% reduction in fasting total and LDL cholesterol in 8 weeks of consumption of RJ can be considered relevant.

Reducing oxidative stress and inflammation is also the goal of many medications and dietary supplements to decrease complications of different metabolic diseases [39]. In the available data, there are some reports confirming the role of RJ as a scavenger of free radicals [21, 37]. Despite antioxidant properties of the RJ found in both* in vitro* and* in vivo* models, there are only a few human studies in patients with type 2 diabetes confirming its effectiveness. Pourmoradian et al. showed a significant decline in malondialdehyde levels and an increase in superoxide dismutase (SOD) and glutathione peroxidase (GPx) activity [40]. Similar results were reported by Shidfar et al., where in addition, an increase in total antioxidant capacity (TAC) was observed [41]. In contrast, our findings revealed a downregulation of (*SOD*) gene in RJ group compared to placebo group, whereas changes in expressions of other analysed genes of oxidative stress pathway (*CAT, GPx, *and* GR*) were not different between groups (RJ* versus* placebo). Although gene of endogenous SOD was downregulated, RJ administration resulted in remarkably higher levels of TAC, bilirubin, and uric acid in serum. As RJ alone displayed very little potency at scavenging any ROS [22], we can speculate that RJ administration improves oxidative stress by enhancing the production of endogenous antioxidants, resulting in higher TAC. The result that 4-hydroperoxy-2-decenoic acid ethyl ester, a RJ fatty acid derivative, markedly induces heme oxygenase-1 expression in cell culture [42] further explains the higher levels of bilirubin, an end product of heme degradation catalysed by heme oxygenase-1 after RJ administration.

In addition to RJ's anti-oxidative effects, our results showed also that the administration of RJ significantly reduced inflammatory marker CRP and significantly increased anti-inflammatory adiponectin. The results are in line with study of Khoshpey et al. [17] where IL-6 was remarkably reduced after supplementation with 3000 mg/day RJ for eight weeks. In addition, our results are also in agreement with the study of Yoshida et al., where the increase in expression of adiponectin in visceral fat and adiponectin receptor 1 in liver in mice was linked to the increase in phosphorylated AMP-activated protein kinase (pAMPK), peroxisome proliferator-activated receptor-*α* and peroxisome proliferator-activated receptor gamma coactivator 1-*α* [32]. These three proteins could reduce inflammation and oxidative stress and improve lipid utilization [43, 44], all of which were also observed in our study. This is therefore a possible mechanism of the profound increase in serum adiponectin level after RJ supplementation.

One of the most important goals in prevention of chronic noncommunicable diseases is improving glycaemic status and maintaining it in the normal range [45]. Recent studies of the effects of supplementary RJ on glucose metabolism have been contradictory. In the present study, fasting glucose was significantly lower at week 8 than at baseline in the RJ group, but there was no significant difference in changes between the two groups at the end of the intervention. We have to stress that our participants were subjects with normal basal glucose levels. In addition, our participants were given a dose of 666 mg of lyophilised RJ before breakfast. Therefore, the reasons why we could not obtain significant effect on fasting glucose are smaller doses of RJ and normal basal glucose levels in our study.

Regarding anthropometric measures, 8 weeks intervention with RJ or placebo did not exhibit any major differences in body fat and fat free mass; therefore, all above mentioned changes in inflammatory, antioxidant, and lipid parameters are not due to changes in body composition nor energy intake. However, a small but significant reduction in BMI and a significant increase in phase angle were observed in follow-up period. A significant increase in phase angle in RJ group compared to placebo group could be connected to health-promoting properties of RJ, because phase angle is a sensitive index of body cell mass and electrical integrity of vital cell membranes [46]. Results of previous clinical studies regarding effects of RJ on weight management are contradictory. Pourmoradian et al. [33] showed a reduction in body weight of diabetic patients after RJ supplementation, and they attributed it to increased oxygen metabolism and oxidative phosphorylation. On the other hand, Guo et al. demonstrated that intake of 6 g of RJ does not show any substantial changes in the levels of body weight, fat, and waist circumference [37]. In the present study, a small decrease in BMI at follow-up in RJ group could be also the consequence of increased oxygen metabolism and oxidative phosphorylation and significant increases in serum leptin levels. This hypothesis is further supported by the fact that regardless of the decreased appetite, the participants did not decrease the total energy intake or the intake of any macronutrient. It is known that leptin stimulates AMPK activation in skeletal muscle but reduces AMPK activity in hypothalamus [47]. Hypothalamic AMPK modulates the functions of different neuronal populations, thereby controlling appetite and energy balance [48]. Decrease in appetite of our participants in RJ group could be explained by the action of leptin and reduced AMPK activity in hypothalamus. Regarding the positive relationship between leptin and adiponectin in the present study, recently, it was demonstrated that leptin upregulates adiponectin expression; and, that the decreased adiponectin expression in established obesity may be secondary to impairment of leptin signalling [49]. Perhaps, RJ administration restored leptin signalling in our participants and, therefore, increases in adiponectin levels were observed. The action of leptin in the RJ group could be connected also to reduced negative mood. In animal models, Lu et al. [50] reported that leptin had an antidepressant-like activity, and it is known that leptin is decreased in the depressive patients[51]. It has been also reported that RJ reduces anxiety in hyperactive subjects [52]. Which component of RJ is responsible for observed effect is not known yet.

Regarding the limitations of our study, we can point to the duration of supplementation (8 weeks). Second, we do not know the most effective dose of RJ for a human being. Larger or smaller daily amounts of RJ may have been necessary to produce some important effects. RJ is characterized by very complicated biological matrix and its composition varies depending on many factors, which are sometimes very difficult to control. Moreover, in the present study, we did not observe any serious adverse effects of RJ. However, increased consumption of RJ in health food supplements may increase incidence of RJ-related allergic reactions [53].

## 5. Conclusions

In conclusion, the administration of 666 mg of lyophilised RJ per day resulted in beneficial effects on lipid profile, inflammation, oxidative stress, negative mood, and satiety in asymptomatic overweight adults. However, further investigations are needed to determine the duration and effective dosages of RJ supplementation.

## Figures and Tables

**Table 1:** Baseline characteristics of asymptomatic subjects included in clinical trial.

	Placebo group	Royal jelly group	p value
Gender (% F, M)	63% F, 37% M	60% F, 40% M	0.964
Age (years)	41.1±7.4	41.1±11.8	0.998
Body fat (%)	28.0±5.6	28.8±9.2	0.773
BMI (kg/m^2^)	27.7±3.7	26.2±1.7	0.415
Total cholesterol (mmol/L)	5.60±1.11	5.65±1.74	0.932
Fasting glucose (mmol/L)	5.66±0.42	5.53±0.78	0.615

*Abbreviation.* BMI: body mass index; F: female; M: male.

Values are expressed as means ± SD. p-value denotes difference between groups using an independent samples t-test.

**Table 2:** Baseline characteristics of asymptomatic subject's energy and macronutrient intake.

	At baseline		Week 8	
	Placebo group	Royal jelly group	Placebo group	Royal jelly group
Energy intake (kcal)	2063 ± 778	1969 ± 613	2020 ± 750	2049 ± 653
Proteins (g)	86.7 ± 23.1	85.8 ± 42.7	86.8 ± 22.8	86.3 ± 42.2
Carbohydrates ( (g)	195.2 ± 114.7	221.4 ±75.9	196.4 ± 114.7	220.9 ±75.7
Fats (g)	95.9 ± 37.4	80.2 ± 25.2	96.8 ± 37.2	79.8 ± 25.0

Values are expressed as means ± SD.

**Table 3:** Anthropometric parameters in asymptomatic subjects before and after intervention.

	Body fat (%)		Fat free mass (kg)		BMI (kg/m^2^)		Phase angle (°)	
	Placebo	Royal jelly	Placebo	Royal jelly	Placebo	Royal jelly	Placebo	Royal jelly
Initial	28.0±5.6	28.8±9.2	60.9±11.8	56.0±13.2	27.7±3.7	26.2±1.7	6.41±0.43	5.94±0.75
Week 4	27.8±5.4	27.6±9.7^b^	61.0±11.3	55.8±13.0	27.6±3.4	26.1±1.8	6.50±0.48	6.05±0.81^b^
Week 8	27.5±5.4	28.0±9.1	60.8±11.2	53.9±11.5	27.7±3.2	26.1±1.7	6.31±0.56	6.02±0.73^a, b^
F/U	27.8±5.2	26.8±9.8^b^	60.9±10.0	55.1±12.3	27.9±3.1	25.8±1.8^a, b, c^	6.29±0.50	6.11±0.71^a, b^

*Abbreviations.* BMI: body mass index; F/U: follow-up.

Values are expressed as means ± SD.

^a^ p-value denotes significant (p<0.05) difference between the changes from baseline on royal jelly vs. placebo.

^b^ p-value denotes significant (p<0.05) difference as compared with the initial within group using a paired t-test.

^c^ p-value denotes significant (p<0.05) difference between the two groups (royal jelly vs. placebo) at a given time point.

**Table 4:** The metabolic profile in asymptomatic subjects before and after intervention.

	TC (mmol/L)		LDL (mmol/L)		HDL (mmol/L)		TAG (mmol/L)		Glucose (mmol/L)	
	Placebo	Royal jelly	Placebo	Royal jelly	Placebo	Royal jelly	Placebo	Royal jelly	Placebo	Royal jelly
Initial	5.60±1.11	5.65±1.74	3.99±1.04	3.72±1.44	1.55±0.25	1.83±0.51	1.38±0.28	1.09±0.45	5.66±0.42	5.53±0.78
Week 4	5.57±1.07	5.38±1.08^a, b^	3.92±0.97	3.59±1.03	1.50±0.29	1.80±0.44	1.31±0.28	0.99±0.47^b^	5.83±0.28	5.32±0.54
Week 8	5.58±0.81	5.27±1.29^a, b^	3.92±0.76	3.55±1.23	1.47±0.28	1.77±0.42	1.39±0.44	1.01±0.50	5.60±0.48	5.22±0.55^b^
F/U	5.56±1.02	5.21±1.70^a, b^	4.05±1.24	3.49±1.07^a, b^	1.42±0.29	1.75±0.39	1.40±0.38	1.02±0.67	5.58±0.56	5.25±0.56

*Abbreviations.* F/U: follow-up; HDL: high density lipoproteins; LDL: low density lipoproteins; TAG: triacylglycerols; TC: total cholesterol.

Values are expressed as means ± SD.

^a^ p-value denotes significant (p<0.05) difference between the change from baseline on royal jelly vs. placebo.

^b^ p-value denotes significant (p<0.05) difference as compared with the initial within group using a paired t-test.

**Table 5:** Inflammatory and anti-inflammatory markers in asymptomatic subjects before and after intervention.

	CRP (*μ*g/L)		Adiponectin (*μ*g/mL)		Bilirubin (*μ*mol/L)		UA (*μ*mol/L)	
	Placebo	Royal jelly	Placebo	Royal jelly	Placebo	Royal jelly	Placebo	Royal jelly
Initial	1.25±0.54	1.59±0.66	12.0±6.3	12.8±5.8	12.0±5.3	7.8±4.5^c^	346±76	294±84
Week 4	1.37±0.83	1.36±0.52	11.8±6.4	13.6±6.4	9.2±4.9^b^	9.6±5.0^a, b^	293±69^b^	293±60^a^
Week 8	1.32±0.76	1.28±0.42^a, b^	8.9±6.5	17.2±6.7 ^a, b, c^	9.4±6.0^b^	10.5±5.2^a, b^	302±53^b^	310±74^a^
F/U	1.48±0.73	1.25±0.31^a, b^	8.5±5.1	16.7±6.8^a, b, c^	8.0±4.3^b^	8.2±4.7^a^	320±70	316±57^a^

Abbreviation: CRP, C-reactive protein; F/U, follow up; UA, uric acid.

Values are expressed as means ± SD.

^a^ p-value denotes significant (p<0.05) difference between the change from baseline on royal jelly vs. placebo.

^b^ p-value denotes significant (p<0.05) difference as compared with the initial within group using a paired t-test.

^c^ p-value denotes significant (p<0.05) difference between the two groups (royal jelly vs. placebo) at a given time point.

**Table 6:** Hormones in asymptomatic subjects before and after intervention.

	Leptin (ng/mL)		BDNF (pg/mL)		NPY (pg/mL)		Cortisol (*μ*g/dL)	
	Placebo	Royal jelly	Placebo	Royal jelly	Placebo	Royal jelly	Placebo	Royal jelly
Initial	53.8±36.3	56.6±40.5	1823±495	1712±674	24.4±6.7	27.2±5.9	3.25±2.10	3.17±1.58
Week 4	52.8±34.4	60.8±42.4	1680±480	1680±610	25.8±7.4	26.8±6.4	3.58±1.84	3.46±1.81
Week 8	49.9±31.9	66.3±45.8^a, b^	1812±476	1797±625	35.4±7.5^b^	31.3±6.5	3.23±1.77	3.57±1.84
F/U	49.3±29.3	64.0±46.2^a^	1842±510	1865±895	30.0±8.0^b^	33.6±5.1	3.52±2.00	3.59±1.60

*Abbreviations.* BDNF: brain-derived neurotrophic factor; F/U: follow-up; NPY: neuropeptide Y.

Values are expressed as means ± SD.

^a^ P-value denotes significant (P<0.05) difference between the change from baseline on royal jelly vs. placebo.

^b^ P-value denotes significant (P<0.05) difference as compared with the initial within group using a paired t-test.

**Table 7:** Mood and appetite in asymptomatic subjects before and after intervention.

	Positive affect		Negative affect		Appetite	
	Placebo	Royal jelly	Placebo	Royal jelly	Placebo	Royal jelly
Initial	34.7±4.0	29.3±11.5	21.1±5.5	19.1±9.8	13.3±3.2	15.4±3.1^c^
Week 4	33.7±13.6	29.8±12.2	18.8±8.1	17.0±8.5	12.9±3.5	13.8±3.2^b^
Week 8	31.7±7.5	30.0±10.0	22.3±6.9	16.0±11.2^b^	13.1±2.7	13.9±3.2
F/U	32.1±4.6	32.0±9.0	23.5±5.5	16.5±6.1	13.5±3.6	13.9±2.8^b^

*Abbreviations.* F/U: follow-up.

Values are expressed as means ± SD.

^b^ p-value denotes significant (p<0.05) difference as compared with the initial within group using a paired t-test.

^c^ p-value denotes significant (p<0.05) difference between the two groups (royal jelly vs. placebo) at a given time point.

## Data Availability

The data (participant's measures) used to support the findings of this study are available from the corresponding author upon request.

## Supplementary Materials

Supplementary Materials Graphical abstract for effects of royal jelly administration on lipid profile, satiety, inflammation, and antioxidant capacity in asymptomatic overweight adults.
