# Supplementary figures and images for: Effects of Royal Jelly Administration on Lipid Profile, Satiety, Inflammation, and Antioxidant Capacity in Asymptomatic Overweight Adults
Source: Evid Based Complement Alternat Med. 2019 Jun 13;2019:4969720. doi: 10.1155/2019/4969720 (PMC6595335; doi:10.1155/2019/4969720)

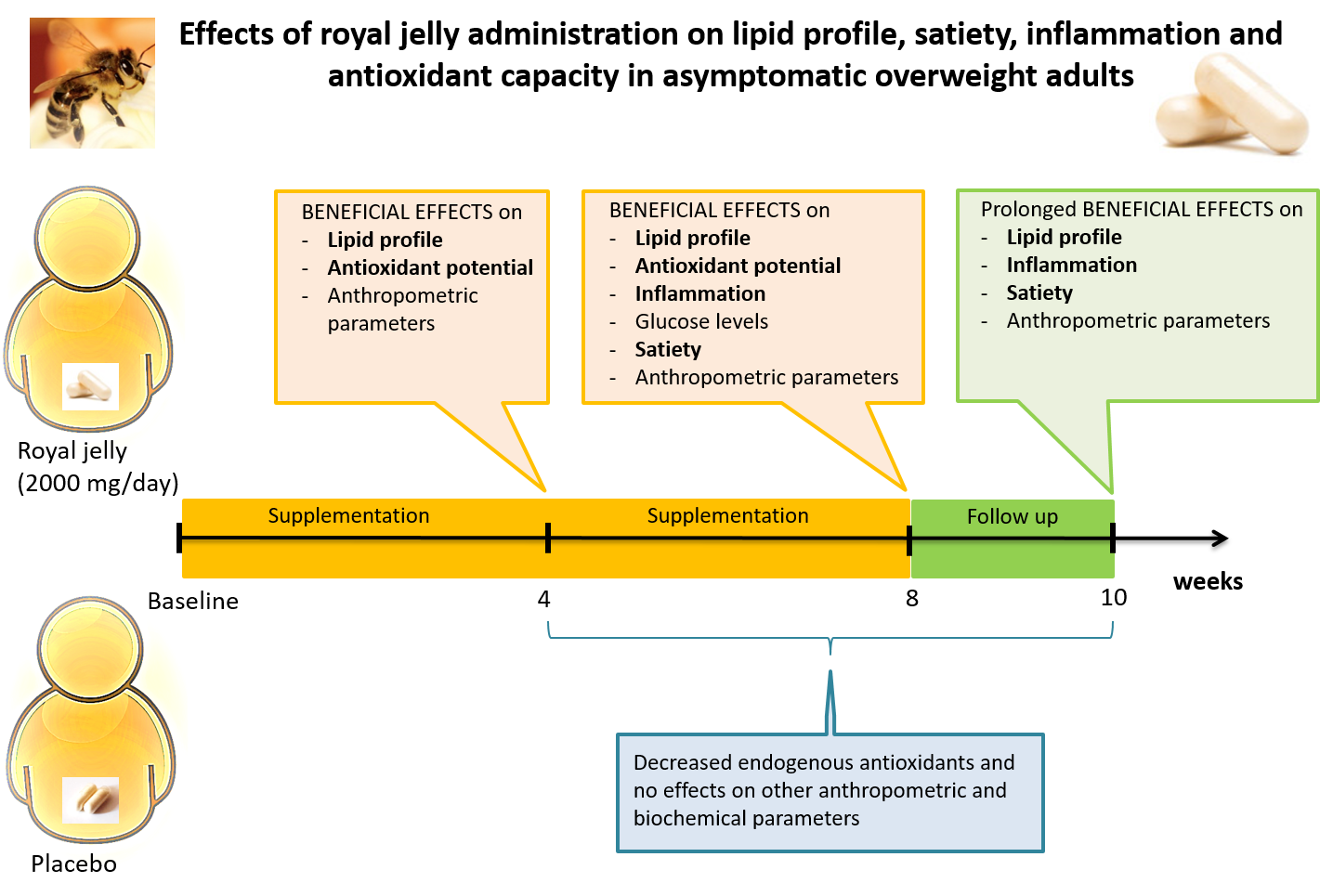

Supplement: Supplementary Materials — Graphical abstract for effects of royal jelly administration on lipid profile, satiety, inflammation, and antioxidant capacity in asymptomatic overweight adults. [file 4969720.f1.tif]
